# Supplementary material for: Hepatopulmonary syndrome has low prevalence of pulmonary vascular abnormalities on chest computed tomography
Source: PLoS One. 2019 Oct 18;14(10):e0223805. doi: 10.1371/journal.pone.0223805 (PMC6799931; doi:10.1371/journal.pone.0223805)
Supplement: Attachment — Submitted filename: Response to Reviewers.docx [file pone.0223805.s002.docx]

September 20^th^ 2019

Joerg Heber

Editor-in-Chief

*Plos One*

Dear Editor:

I wish to submit an revised manuscript for publication in the *PLOS ONE* , titled “Hepatopulmonary syndrome has a low prevalence pulmonary vascular abnormalities on chest computed tomography.” Felipe S.Torres, Juliana F. Zampieri, Betina C. Machado, Marli M. Knorst and Marcelo B. Gazzana coauthored the paper. Bellow the modifications required.

- Please ensure that your manuscript meets PLOS ONE's style requirements, including those for file naming.

The manuscript was reviewed to meet PLOS ONES style requirements.

- Reviewer #1: The title is a bit misleading as it seems to suggest a relationship between HPS and pulmonary vascular abnormalities. The title could be re-worded to more accurately reflect the findings.

Thank you for this comment. The title was modified to “Low prevalence of	pulmonary vascular abnormalities on chest computed tomography in patients with	hepatopulmonary syndrome.”

- Reviewer #1: Abstract is well written and appropriately reflects the remainder of the submission. Some changes are required. In the second sentence of the abstract – please change “access” to “assess”. All locations that have O2 in the abstract and main text should have the 2 as a subscript. Currently, they are all superscript. Finally, the first sentence of the conclusion should state “The prevalence of pulmonary…”

Thank you for all observations. All suggestions were modified accordingly	and the abstract was appropriately corrected, as follows:

“This study aimed to assess the prevalence of type 1 and 2 pulmonary	vascular abnormalities on chest computed tomography (CT)”

“The prevalence of pulmonary vascular abnormalities on chest CT of	patients with cirrhosis and HPS is low and not correlated with PaO2.”

- Reviewer #1: The first paragraph under “Patients” needs some re-wording. The second sentence in the first paragraph in particular seems incomplete and reads awkwardly. It appears the authors are trying to state how the patients were selected, but the sentence is a fragment. In addition, the first criteria (i) seems too vague. Presence of liver disease is a wide ranging statement and could represent acute or chronic. The authors should be more specific here (e.g. chronic liver disease with portal hypertension).

“Patients were included where they had a chest CT and a diagnosis of HPS	based	on the following criteria: (i) presence of chronic liver disease; (ii)	alveolar–arterial	oxygen gradient (AaO2) >15 mmHg (20 mmHg in patients	over 64 years old) detected	with blood gas analysis; and (iii) demonstration of	intrapulmonary vascular dilatation by means of a positive contrast-enhanced	echocardiography or perfusion lung scanning with technetium-99m-labelled	macroaggregated albumin (1,2.)”.

- Reviewer #1: A clarification is required, though. In the paragraph that begins “There was no difference in the prevalence of pulmonary vascular abnormalities when comparing normoxemic (n=20) and hypoxemic patients (n=18) (p=0.513),” the final sentence is incomplete. Presumably, the authors were trying to state there was no correlation between PaO2 and ABR; however, please adjust accordingly.

“There was no difference in the prevalence of pulmonary vascular abnormalities	when comparing normoxemic (n=20) and hypoxemic patients (n=18) (p=0.513).	The mean PaO2 of patients with any vascular pulmonary abnormality on CT	(n=11) was 82.1 mmHg ± 17.9 and for those without any pulmonary vascular	abnormality (n=27) it was 77.9 mmHg ± 15.8 (p=0.515). There was no correlation	between PaO2 and ABR (Figure 3).”

- Reviewer #1: Discussion: The discussion appears thorough and well written. The third to last sentence is written as “The prevalence of these abnormalities on Chest CT is low thanand were not correlated with PaO²,” and requires spelling and grammatical correction.

“The prevalence of these abnormalities on Chest CT were not correlated with PaO2.”
